# Pattern Recognition and Anomaly Detection by Self-Organizing Maps in a Multi Month E-nose Survey at an Industrial Site

**DOI:** 10.3390/s20071887

**Published:** 2020-03-29

**Authors:** Sabina Licen, Alessia Di Gilio, Jolanda Palmisani, Stefania Petraccone, Gianluigi de Gennaro, Pierluigi Barbieri

**Affiliations:** 1 Department of Chemical and Pharmaceutical Sciences, University of Trieste, Via L. Giorgieri 1, 34127 Trieste, Italy; slicen@units.it; 2 Department of Biology, University of Bari “Aldo Moro”, Via Orabona 4, 70126 Bari, Italy; jolanda.palmisani@uniba.it (J.P.); stefania.petraccone@uniba.it (S.P.); gianluigi.degennaro@uniba.it (G.d.G.)

**Keywords:** electronic nose, ambient air, oil and gas, self-organizing map, neural networks

## Abstract

Currently people are aware of the risk related to pollution exposure. Thus odor annoyances are considered a warning about the possible presence of toxic volatile compounds. Malodor often generates immediate alarm among citizens, and electronic noses are convenient instruments to detect mixture of odorant compounds with high monitoring frequency. In this paper we present a study on pattern recognition on ambient air composition in proximity of a gas and oil pretreatment plant by elaboration of data from an electronic nose implementing 10 metal-oxide-semiconductor (MOS) sensors and positioned outdoor continuously during three months. A total of 80,017 e-nose vectors have been elaborated applying the self-organizing map (SOM) algorithm and then k-means clustering on SOM outputs on the whole data set evidencing an anomalous data cluster. Retaining data characterized by dynamic responses of the multisensory system, a SOM with 264 recurrent sensor responses to air mixture sampled at the site and four main air type profiles (clusters) have been identified. One of this sensor profiles has been related to the odor fugitive emissions of the plant, by using ancillary data from a total volatile organic compound (VOC) detector and wind speed and direction data. The overall and daily cluster frequencies have been evaluated, allowing us to identify the daily duration of presence at the monitoring site of air related to industrial emissions. The refined model allowed us to confirm the anomaly detection of the sensor responses.

## 1. Introduction

Currently people are aware of the risk correlated to pollution exposition. Thus odor annoyances generate immediate alarm among citizens because malodor is considered a warning about the possible presence of toxic volatile compounds. Annoyances present can cause stress in the population reducing the quality of life and leading to health side-effects [1,2,3,4]. The problem worsens when people live in proximity of emissive sources and/or when the emissions are mainly fugitive [5,6,7,8]. From the point of view of environmental and industrial olfactory nuisances, impacts come from factors as frequency, intensity, duration, offensiveness, and location of the odor events, on the whole known as FIDOL [9,10]. The time dimension of olfactory pollution is relevant since contacts between odorants in the environment and exposed population can be discontinuous, repeated at irregular intervals, of variable duration, making sampling of transient events impacting on the complaining citizens a relevant and not a trivial task. A continuous monitoring is thus highly preferable for studying malodor phenomena and then managing complaints of citizens. Air samples can be characterized by different methods, recently reviewed in [11], covering both sensorial and instrumental/analytical approaches. Among sensorial ones, dynamic olfactometry (standardized as for instance in the European technical norm EN13725: 2003 [12], under revision), field inspections (as in European technical norm EN16841:2016 [13]) and recording from residents are considered. Most diffuse instrumental/analytical methodologies are gas chromatography–mass spectrometric or olfactometric detectors [14], identification of specific compounds, and electronic noses, named also e-noses or more recently instrumental odor monitoring systems (IOMS) [15]. The first ones allow one to identify and quantify specific odor compounds consisting of air samples. It should be considered that the odor sensation in environmental contexts is produced in general by complex mixtures of chemical compounds, with odor intensity depending on both concentration and odor threshold of the components of the mixtures [16,17], making low concentration components with a low odor threshold (e.g., reduced sulfur compounds) very relevant in determining the sensorial response. 

Electronic noses are useful instruments, responsive to the presence of mixtures of odorant compounds of variable and only partially known composition, at concentrations relevant for human odor sensation (ppb_v_, or low ppm_v_), with a high sampling frequency [18,19,20,21]. Electronic noses are generally constituted by (1) a sampling device, pumping air in a controlled way to (2) a system of sensors producing a multivariate signal after reversible interaction with the mixture of compounds present in the gaseous sample; and (3) multivariate signals are processed identifying patterns related to the presence or absence of odorants and/or to odor intensity and/or to discrimination among odor sources. The system of transductors can be an array several type of sensors as chemoresistors (e.g., metal-oxide-semiconductors (MOS), metal-oxide-semiconductor field effect transistors, (MOSFET), and composite conducting polymer (CCP) sensors), polymer functionalized quartz crystal microbalance (QCM), surface acoustic wave (SAW) sensors, polymer coated fiber-optic evanescent wave (FOEW) sensors, polymer functionalized and micro-electromechanical system (MEMS), cantilever sensors, and surface plasmon resonance sensor (SPR) sensors [22]. Signals of electronic noses [23] can be used in an unsupervised way in order to identify changes in the composition of sampled air, related to gaseous emissions. The process of defining correlations between raw electronic nose data and sensorial or instrumental responses for samples of known origin or composition are obtained, possibly diluted in a controlled way [19], is known as the training phase of the electronic nose. Trained electronic noses allow one to monitor variability in the composition of gaseous samples, defining odor presence/absence or odor concentration estimates, or belonging of an unknown sample to a defined sample class. The use of a trained model in operational conditions needs to account for sensor drift, changes in operation conditions, humidity changes, and the background or matrix effect, among others [20]. The model relating signal patterns recorded for sampled air to signal patterns recorded for samples having a known origin or composition (as the ones collected at emission sources or synthetic ones or samples thoroughly chemically characterized) can be supervised discrimination or classification models, as produced by linear discriminant analysis (LDA), classification by feed-forward back propagation neural network (BPNN), k-nearest neighbor (K-NN), support vector machine (SVM), and random forest (RF) [19,22,24]. 

In the variegated range of electronic noses applications, as pattern recognition and identification of sparse samples or quality control in productive cycles, a particular, challenging and relevant task is environmental odor monitoring in the presence of industrial plants with variable emissions, where the training phase hardly can exhaustively cover/identify training samples for all possible states of the open environmental system under study. Moreover, the samples from odor emission sources from private companies are not always available so that supervised discrimination and classification is not always possible. In these cases, an unsupervised approach can provide useful information for detailing relevant variability and dynamics of composition related to the presence of odors from specific source emission at receptor sites [25,26].

Electronic noses applied for environmental monitoring are often modifications of instruments designed for applications in controlled conditions, as food or a product quality check performed in laboratories or indoor air monitoring. Environmental monitoring for relatively long periods (e.g., several months to one year) and in close proximity of odor emission sources, requires checks for appropriate functioning of multisensor systems as electronic noses are. Periodic recalibrations and check functioning routines are needed; environmental conditions can be very variable and air severely polluted can be sampled and sensed, possibly driving the sensors system out of an optimal range of responses. At present instrumental approaches and procedure for identifying the presence of odorant mixtures with high temporal resolution are not standardized, and studies highlighting operational pitfalls and solutions are required to define successful strategies of characterization of complex situations in open systems.

With the aim of characterizing temporal evolution of odor nuisances close to industrial plants with relevant fugitive emissions, a method called the odor control map (OCM) has been proposed for compression, visualization, clustering of up more than hundred thousand vectors of signals produced by e-noses. This is a two steps method, based firstly on modeling the e-nose data by the neural network algorithm for unsupervised pattern recognition known as the self-organizing map (SOM), and then using k-means clustering on SOM outputs [25]. In this application, the SOM algorithm operates on vectors of outputs of the n sensors of the e-nose, allowing one to identify recurrent patterns (or air type profiles or neuron of map) that are organized in a bidimensional map, accordingly to the similarity among neurons in the n-dimensional space, allowing the visualization and assessment of odor-related measured features at the monitoring site, discussing synoptically also discontinuous instrumental and sensorial measurements. Up to now, studies have been published on the characterization of odor impacts close to an integral cycle steel plant during four months monitoring [25], and on biowaste composting during seventy days survey [26], both with the same type of hybrid e-nose, implementing an array of nanocomposite sensors, MOS and electrochemical sensors.

In this paper we present an assessment of the evolution of air quality by means of a two stage odor control map approach, applied to patterns of sensor signals of a commercial electronic nose implementing ten MOS sensors, in proximity of a gas and oil pretreatment plant, characterized by fugitive emissions [27,28] hardly described by dispersion modeling, and object of attention for health impacts assessment on population of the surrounding area [29,30]. The clusterization of the OCM neurons leads to the identification of a preliminary number of air types measured by the electronic nose. In the present study we illustrate an example of the anomaly detection capabilities of the method, based on visual inspection of the distribution of cluster share within monitoring days. A two stage SOM and k-means strategy is exemplified—leading to retaining only the part of the overall data set related to relevant air quality variations—and thus to a build better balanced OCM model, confirming anomalous e-nose recording possibly related to instrument malfunctioning or to the emerging absolute dominance of one of the experimentally detected odor patterns.

## 2. Materials and Methods

### 2.1. Site Description

**Figure 1:**
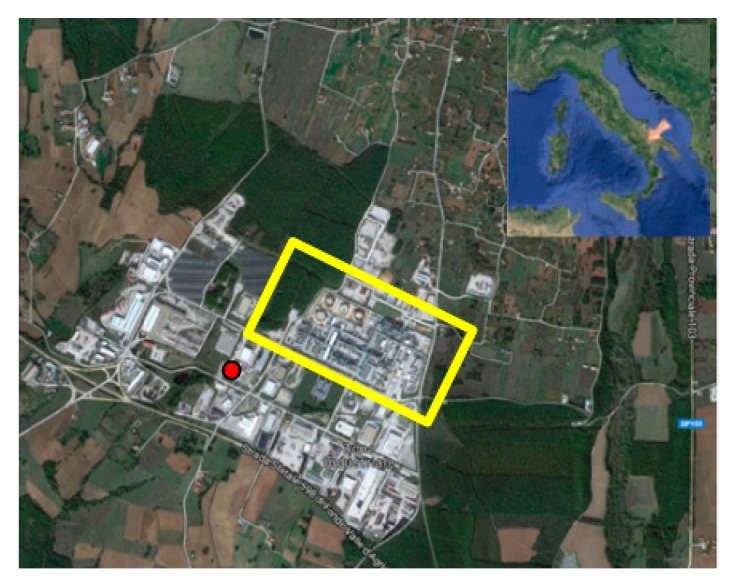
Map of the monitored site. The red dot shows the position of the e-nose; the yellow rectangle shows the position of the plant. On the upper right of the figure, an inset with the position of the site on the Italian territory is shown by an arrow.

One of the biggest crude oil and gas onshore European reservoirs is placed in Val d’Agri (South of Italy). The Val d’Agri and more specifically the industrial area of Viggiano hosts, in a rural and populated area, 24 oil wells the largest existing oil/gas pre-treatment plant (surface 170,000 m^2^ with different odor emission sources) denominated Centro Olio Val d’Agri—COVA (40.68°N, 15.53°E). The industrial area is characterized by fugitive emissions due to leaks from the tanks and/or the extraction process and to system over-charging, with temporal and chemical variability, characterized by different classes of compounds such as aromatic and aliphatic hydrocarbons, sulphur-compounds having low odor threshold values and non-methane volatile organic compounds. The integration of different technologies able to real time monitor volatile organic compounds (VOCs) and odor compounds with respect to wind speed and direction, results in the best synergistic approach to identify and characterize the emission sources. Therefore, in this study an odor monitoring campaign was conducted from the 7th April 2017 to 28th June 2017 at a monitoring site positioned approximately South-West to COVA (Figure 1). The monitoring station remotely controlled through the GSM-wireless network, consisted of an electronic nose (e-nose), a photoionization detector (PID) and a weather station and it was able to collect real time data about odorant mixture profiles, VOC concentrations, temperature, relative humidity, wind speed, and direction.

### 2.2. Electronic Nose Continuous Monitoring

An electronic nose is an instrument composed of an array of electronic chemical sensors with partial specificity and a pattern recognition system, which is able to recognize also a complex mixture of odorants [3]. The instrument used in this study was PEN3 by Airsense Analytics GmbH (Schwerin, Germany) with (Win Muster software v.1.6) for data management. The sensor array is composed of ten different thermo-regulated (150–500 °C) metal oxide semiconductors sensors (MOS) that are cross-sensitive to molecules belonging to several chemical classes or having diverse functional groups. In fact, based on molecules chemical characteristic, an oxygen exchange between the volatile gas molecules and the metal coating material occurs. As a consequence, changes in the electrical resistance of sensors were registered and elaborated in order to generate a pattern corresponding to the gas composition of the air sample. Moreover, because sensor conductivity drifted as the sample gas passed over the array, the data were adjusted based on the changing ratio of conductivity between G and G_0_ (i.e., the electrical conductivity response of the sensors to the sample gas relative to the carrier gas or baseline signal over time). Therefore, prior to the data collection, the zero gas (air filtrated by active carbon) was pumped at the backside of the instrument into the electronic nose at a rate of 600 mL/min in order to rinse the metal oxide gas sensors from unwanted gas particles, which can contribute to a reading error. During the monitoring phase the air was continuously sent to the sensor chamber (whose volume was about 150 mL) by pump through the inlet at a rate of 400 mL/min. Measurement cycles were set at 10 s of zeroing and 50 s of sampling. Data from the sensor array were then collected per-minute obtaining a vector of 10 values for every minute. 

In this study, the sensors are named by acronyms from S1 to S10, and the sensor features as stated by the manufacturer are reported in Table 1.

Before the start of the monitoring campaign, a calibration procedure was performed by the manufacturer and Quality Assurance/Quality Control procedures (QA/QC) revealed a Limit of Detection (LOD) ranging from 0.1 to 5 ppm for gases and organic solvents. In addition, during the monitoring campaign an in-field calibration was conducted biweekly.

### 2.3. Total Volatile Organic Compounds (VOCs) Continuous Monitoring

The PID was demonstrated as a rapid, effective instrumental technique to evaluate volatile organic compounds (VOCs) concentrations and human VOC exposures. Therefore, in this study, the real time total VOCs (TVOCs) concentration was monitored by means of a high-time resolution photo-ionization detector (Corvus, Ion Science Ltd, UK). This monitor utilizes high sense technology and a 10.6 eV lamp to detect TVOCs down to low part-per-billion (ppb) levels. In fact, it measures concentrations of TVOC from 0.02 to 20 ppm with a LOD equal to 5 ppb and an accuracy of ±5 ppb. Moreover, Corvus monitor features built-in humidity compensation due to the integrated humidity and temperature sensors, and thus automatically corrects TVOC readings for Relative Humidity (RH). Before the monitoring campaign start, it is factory calibrated against isobutylene and thus, TVOCs concentration is reported as ppm_v_ equivalent of that gas. In addition, during the monitoring campaign an in-field calibration was conducted biweekly. In this study, TVOC concentrations were recorded every 1 second and then, TVOC average values for 1-minute tasks were used for the statistical analyses.

### 2.4. Meteorological Data

Weather Station used in this study was a Vantage Pro2™ wireless weather station (Davis Instruments Corporation) including the Integrated Sensor Suite with a rain collector, temperature and humidity sensors, anemometer, and UV and solar radiation sensors, with an interface for logging weather data and uploading weather information to remote repository. Coherently with data collected by PID and e-nose, information about temperature, RH, and wind speed and direction were downloaded per-minute. Temperature and RH were in the range required by PEN 3 technical requirements (Temperature: 0–45 °C), (Humidity (relative) 5%–95%, non-condensing). 

### 2.5. Self-Organizing Maps and Clustering Analysis

Self-organizing maps (SOMs) are part of the unsupervised neural networks family [31,32,33,34], which encompasses minimum spanning tree SOM, neural gas, growing cell structures and competing SOMs. SOM is characterized by straightforward visualization features that have been proved to be useful in pattern recognition in complex open environments [25,35,36,37].

In brief a self-organizing map is a single layer neural network with neurons defined set along a grid, that is usually bidimensional and rectangular. 

During a model building phase, the experimental input pattern vectors (i.e., a set of sensor data measured at a certain time) were presented to all the neurons (set of weights that are modified after a match with input vectors); distances or mismatches were calculated. The neuron of the map that is most similar to an input pattern vector is the winner (best matching unit, BMU). The winner neuron (BMU) modifies itself (adjusting weights) and lightly neurons in the neighborhood, for increasing similarity to the input data, accordingly to learning rules.

Initialization parameters for the algorithm—described in detail in [31]—can be calculated using heuristic rules introduced by Vesanto [32]. The number of neurons of the map were calculated on the basis of the number of input data vectors to be modeled, and small or big maps can be produced on the basis of the level of detail required (small maps are used to extract more general features of the data set, while big ones represent finer details of the data set in comparison to a regular map). The ratio between side-lengths of the map depends on the ratio of the first two eigenvalues of the covariance matrix of the input data. The training can be performed with sequential or batch algorithms, where different neighborhood function can be chosen (e.g., Gaussian function or bubble function), and learning rate can vary linearly, with power series or by an inverse of the time function. The training is repeated for a number of epochs that depends on the ratio between the number of neurons or map units and the number of input vectors.

After the training phase, neurons on the map were prototypes or recurrent patterns for the data set under study. SOMs produced low-dimensional projections images of high-dimensional data distributions on the map, in which similarities relationships among experimental data were preserved. 

The evolution of a dynamic system can be followed plotting the sequence of BMUs for input vectors from the monitoring interval of interest, defining a trajectory for the system on the map [25].

SOM neurons can be then the object of a cluster analysis, allowing powerful visualization features in structured 2D-maps [37,38], which can be considered control charts spanning the possible states of the system under study as described by features measured by input vectors. In the field of unsupervised classification, several clustering algorithms are available, belonging to families known as partitive algorithms, hierarchical algorithms, density based algorithms, spectral methods, and model based methods, recently reviewed and compared in [39]. Partitive algorithms as k-means are known for low computational cost, and good performances in anomaly detection [40] and data segmentation. Supervised and semisupervised classification algorithms can be implemented if respectively all or part of the data are associated to class labels; in these cases a fusion of multiple classifier can lead to improved classification performance [41]. The issue of identification of an appropriate number of clusters, among the number of possible partitionings, is addressed computing validity indexes [42] as the Davies–Bouldin index [43], which compares S_c_ within-cluster distance and d_ce_ between clusters distance. Other effective validity indexes are the Calinski-Harabasz index and the Gamma index [44]. The best partitioning for the Davies–Bouldin index—indicating a partitioning generating compact and well separated clusters—is the one for *K* number of clusters, which minimizes:

(1) $$DB=\frac{1}{K\;}\overset{K}{\underset{i=1,\;i\neq j}{\sum }}\max\left\{\frac{d_{i}+d_{j})}{d\left(c_{i},c_{j}\right)}\right\}$$

with *K* the number of clusters, *d_i_* and *d_j_* the average distances of all objects in a cluster from the correspondent cluster center *c_i_* and *c_j_* and *d(c_i_, c_j_ )* the distance between cluster centers.

The second level of abstraction provided by clustering the SOM units leads to a high level of generalization of features of the data set, that in general terms are easier to interpret than details of specific cases and that can be useful in the rationalization of complex systems as highly dynamical open environments are. Cluster membership of BMUs is transferred to the related original input vectors recorded at defined monitoring times. Parameters measured by different instruments or events recorded at monitoring times belonging to such a cluster, can be clustered consequently, allowing fusion of heterogeneous data. Details on how this algorithm is applied for odor impact assessment in the present work can be found in [25,26]. 

In this paper the temporal analysis of the daily share of the cluster membership will be used for detecting an anomaly in monitoring results that had not been diagnosed by instrumental periodic calibration procedures.

### 2.6. Calculations

SOM calculations and clustering classification were performed in the R software environment [45] implemented by the kohonen package [45,46]. SOM initialization parameters and k-means clustering were performed translating into R language the functions present in SOM toolbox for MATLAB by Vesanto [32]. SOM outputs exploration and SOM visualization were performed using in-house scripts and “openair” package functions [47].

## 3. Results and Discussion

### 3.1. Exploratory Model of E-nose Data

**Figure 2:**
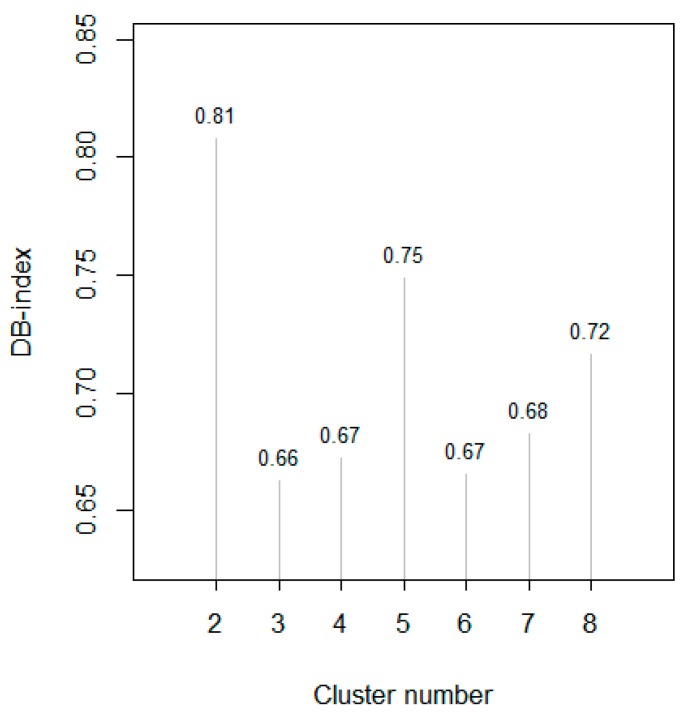
Davies–Bouldin (DB) indexes for cluster number ranging from 2 to 8.

We firstly ran a model using all the data recorded during the whole period: 80017 min recordings for the ten sensors of PEN 3 e-nose were collected from 7th April 2017 to 28th June 2017 at the monitoring site close to COVA. Data were processed to build a SOM parameterized accordingly to heuristic rules presented in [32], with the batch algorithm, Gaussian neighborhood, learning rate that varies by inverse of time, and two learning epochs, producing “small” maps of 30 neurons × 12 neurons, thus identifying 360 prototype vectors of the ten modeled variables, ordered topologically by similarity on the map. With the aim of achieving a further level of abstraction/generalization, the resulting 360 vectors of the map were clustered by the k-means algorithm. The best partition of the prototype vectors was identified after examination of the Davies–Bouldin indexes for 2–8 clusters reported in Figure 2. 

The partition with three clusters was identified as the one showing the lowest Davies–Bouldin index (0.66) among those with number ranging from 2 to 8, thus producing the most compact and well separated groups of data.

**Figure 3:**
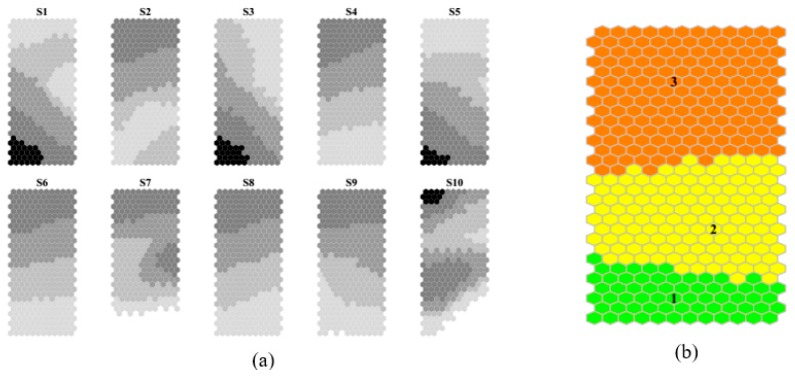
(**a**) Patterns of responses of the ten sensors as planes of the self-organizing map (SOM) of the complete data set. The grayscale indicates clear colors for low values to darker ones for high values of prototype weights; (**b**) localization of clusters on the SOM of the complete data set.

In Figure 3 the distributions of weight values for each sensor on the SOM map are shown as sensor planes of map (a) as well as the cluster splitting of map (b). Similarities among patterns of weights of group of sensors were found as S1, S3 and S5 or S4, S6 and S8. Clusters splits prototypes in clearly defined areas of the map.

**Figure 4:**
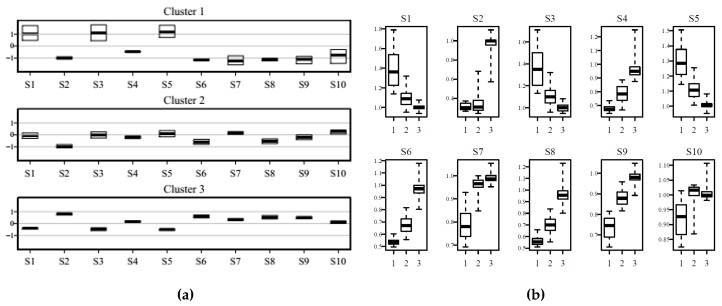
(**a**) Profiles of interquartile ranges and medians of sensor weights for prototypes of the overall dataset belonging to each cluster; variables normalized by sensor and (**b**) boxplots of variability of responses of prototypes of the overall data set for the three clusters for each of the ten sensors

Interval of values assumed by sensor variables for prototypes in each cluster can be visualized as shown in Figure 4, where profiles of interquartile ranges of values of sensor outputs for prototypes belonging to each cluster are reported in (a) and variables were normalized by a sensor to fit in the same y-range, while in (b) boxplots of variability of responses for the three clusters are shown for each of the ten sensors. Variances of clusters 1, 2, and 3 were respectively 145.6, 120.1, and 86.2.

**Figure 5:**
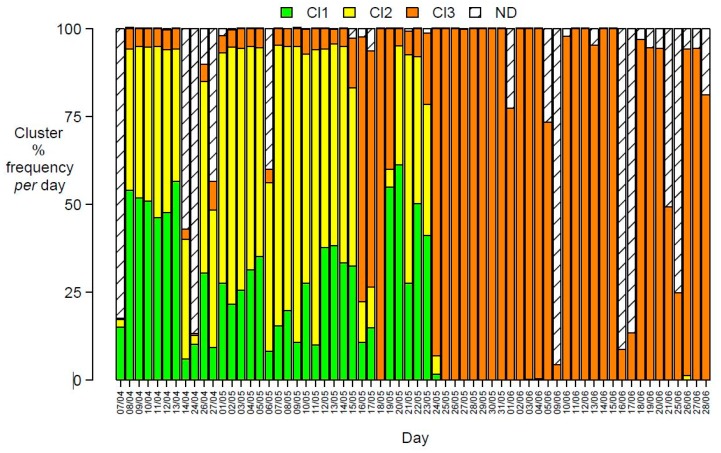
Daily cluster percentage distribution (ND = data not recorded, due to power failures) obtained by the SOM exploratory model for days from 7 April 2017 to 28 June 2017.

It can be seen from both Figure 4a,b how the boxplots for values from prototypes of each sensor of cluster 3 were very narrow, showing very small variability of profiles within this cluster, pointing at the presence of very similar prototypes in it. This characteristic contrasts with relatively high variability for sensor values of prototypes of cluster 1, while those of cluster 2 had intermediate ranges of values. Cluster 3 contained a relevant share of the total number of prototypes, as it can be seen from Figure 3b. In order to characterize this peculiar characteristic of cluster 3, temporal patterns were considered: the cluster percentage share of daily minutes is reported in Figure 5.

Figure 5 shows that, after an initial period in which daily changes spanned the three clusters, data recorded from 25th May 2017 to the end of the monitoring period exclusively belonged to cluster 3, with minimal variability. During the monitoring days, power failures have interrupted data recordings, thus generating some incomplete acquisition of daily data, with a data gap between April 14th to April 24th and in occasional shorter intervals, represented by ND in Figure 5. The permanent change in the cluster share after May 24th, leading to a unique membership of all the electronic nose data to cluster 3, pointed at the occurrence of a sudden critical event rather than to a simultaneous progressive drift of responses of all the 10 sensors. This can be caused by a permanent failure in the e-nose sensor system, undiagnosed by ordinary quality control procedures. A severe change of the emission pattern in the area is considered improbable, due to the minimal variability of cluster 3 data (see paragraph 3.5).

### 3.2. Refined SOM Model 

With the aim of excluding an uncontrolled bias in the model of air composition variability, data from 7th April 2017 to 24th May 2017 were used in the building phase of a new SOM model. Thus from now on, the data used to build the model will be named as “building dataset” and the other data will be named as “external dataset”. Moreover, PID data and wind speed and direction data, which has not been used to build the model will be named as "ancillary data" and used to interpret cluster profiles.

SOM algorithm was run on the building dataset using the batch algorithm, Gaussian neighborhood, learning rate that varies by an inverse of time, and two learning epochs, producing “small” maps; a refined model having 264 prototypes in a 22 × 12 map was obtained; Davies–Bouldin indexes for k-means models with 2–7 clusters are respectively 1.02, 0.89, 0.75, 0.87, 0.76, and 0.78; the smallest one refers to the partition into four clusters, generating compact and well separated clusters.

**Figure 6:**
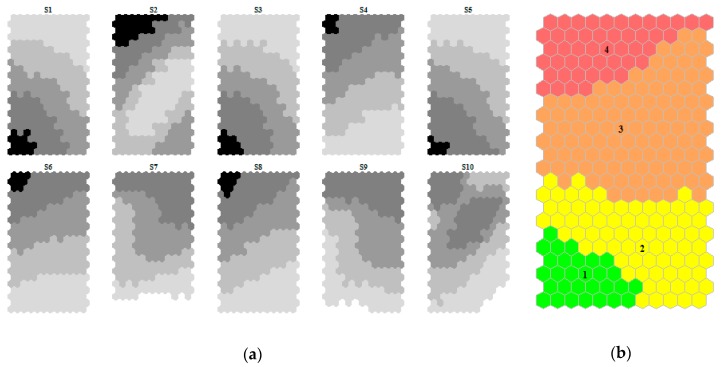
(**a**) Patterns of modeled responses of the ten sensors for the building data set as SOM planes. The grayscale indicates clear colors for low values to darker ones for high values and (**b**) localization of the cluster on the SOM.

Prototype vectors, each with a weight for each of the ten sensors, can be visualized by displaying planes of weights of each sensor, which can be considered as layers of the SOM. A further top layer can be used for displaying further relevant information, as a cluster membership of each prototype. In Figure 6a,b SOM planes with the distribution of weights for each of the ten sensors and the cluster splitting on the map are reported respectively.

Figure 6a shows qualitatively how SOM planes referring to the ten sensors can be grouped according to similar patterns: S1-S3-S5 (sensors named Aromatic1, Aromatic2, and Arom-aliph), S2-S4-S6-S8 (sensors named Broad-range, Hydrogen, Broad-methane, and Broad-Alcohol), and S7-S9 (named Sulphur-organic and Sulphu-chlor). S10 (Methane-aliph) shows a pattern different from the previous ones. Considering that the first group of sensors recorded the signal as G0/G (see Table 1) we could say that both the first and second group of variables show a similar behavior. In general, the lower side of the map was characterized by low values and the upper side by high values of sensor response. Clusters from 1 to 4 were positioned on the SOM in sequence from the bottom to the top side (Figure 6b).

**Figure 7:**
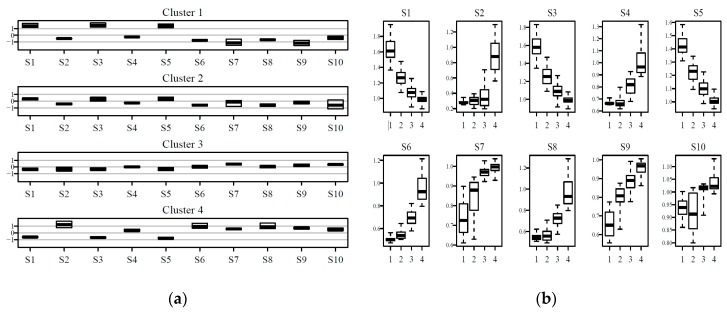
(**a**) Profiles of interquartile ranges and medians of sensor weights for prototypes of the building data set, belonging to each of the four clusters; weights are normalized by sensor; and (**b**) boxplots of weights of prototypes of the building data set, for the four clusters for each of the sensors

Cluster variability of sensor responses of SOM prototypes can be visualized in two ways: by cluster or by sensor, as reported in Figure 7a,b respectively. In Figure 6a the values were normalized for each sensor, in order to fit in the same y-range. 

Looking at Figure 7 the distribution of weights for each sensor and for each cluster can be observed in a detailed way. The characterization of clusters in term of sensor responses can be examined in Figure 7a, with profiles that are reported from cluster 1 to cluster 4 from the top to the bottom of the inset (a). Ranges of weights in the four clusters for each of the ten sensors are displayed in Figure 7b. Considering how conductance was recorded for the sensors by PEN 3 (Table 1), in Figure 7a cluster 1 shows the lowest responses, thus representing clean air accordingly to the e-nose and clusters 2–4 in general terms have increasing signals with cluster 4 grouping patterns of sensor responses for air richer in compounds detectable by PEN 3. Figure 6b shows for sensor S1–S9 monotone trends for medians from cluster 1 to cluster 4, while S10 had a less clear trend, with cluster 2 covering a wide range of values.

### 3.3. Cluster Characterization by Ancillary Data

**Figure 8:**
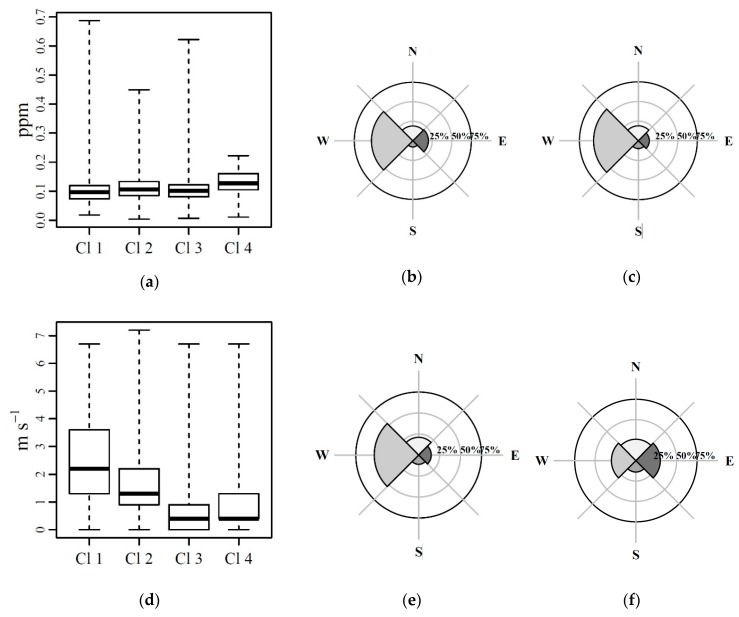
(**a**) Photoionization detector (PID) data distribution according to clusters represented by boxplots; (**b**) wind sector direction percentage for cluster 1; (**c**) wind sector direction percentage for cluster 2; (**d**) wind speed distribution according to clusters represented by boxplots; (**e**) wind sector direction percentage for cluster 3; and (**f**) wind sector direction percentage for cluster 4.

Clusters were characterized by available ancillary data. Cluster membership of a BMU was transferred to the original input vectors mapping on it, which were recorded at defined monitoring times. Parameters measured by different instruments or events recorded at the monitoring times belonging to such a cluster, can be clustered consequently, allowing fusion of heterogeneous data. In other words, if a vector produced by the e-nose at time x maps on a prototype vector p, having membership in cluster c, then a datum produced by instrument i at the same time x will also have a membership in cluster c. TVOC data expressing the VOC concentrations as ppm of isobutylene, and wind speed (m s^−1^) and directions have been considered as ancillary data. TVOC data and wind speed data are represented by boxplots. Wind direction has been represented by the frequency of the wind sector direction (Figure 8). 

In Figure 8a it can be observed that the whole interquartile range of PID concentrations in cluster 4 was higher than the medians of the other clusters. Considering wind directions grouped by sectors, clusters from 1 to 3 (Figure 8b,c,e show a similar distribution in the wind sector frequency, with more than 50% of wind directions at monitoring times grouped in these clusters, blowing from the Western sector. Wind directions at monitoring times grouped in cluster 4 (Figure 8 (f)) had a relatively high percentage of winds from the eastern and northern sectors, which identifies the position of the oil plant with respect to the e-nose (see Figure 1). In Figure 8d, distributions of wind speeds associated to clusters are shown: cluster 1 and 2 had higher median values and distribution of wind speed. Clusters 3 and 4 show similar (relatively low) medians of wind speed but the frequency of wind direction sectors were different (Figure 7e,f). 

Assembling all these outcomes, we could reasonably state that prototypes of cluster 4 represent air samples characterized by e-nose recurrent patterns associated to low wind speeds, wind directions from the COVA plant (northern and eastern sectors), and by relatively high levels of volatile compounds as detected by PID and sensor responses. 

### 3.4. Daily Cluster Distribution 

Cluster distribution of experimental e-nose observation could be evaluated for different time intervals. For the building dataset the overall percentage frequency was: cluster 1—13.4%, cluster 2—26.1%, cluster 3—42.4%, and cluster 4—18.1%.

**Figure 9:**
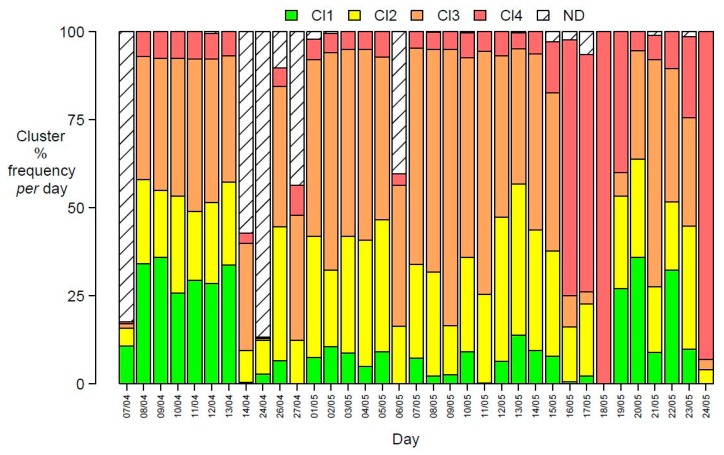
Cluster percentage frequency for each day (ND = data not recorded for power failure).

Daily cluster distributions can be represented as a bar graph as shown in Figure 9. Cluster 4, associated to high e-nose sensor responses had a small daily share (less than 5% of the minutes in a day, slightly more than an hour per day) until May 15th. The daily share of cluster 4 rose, until reaching the whole day on May 18th, then returning to smaller daily shares for three days and then regaining dominance and then exclusivity after May 24th. The pattern recognition ability of the e-nose system for ambient air in the industrial area close to the oil/gas pretreatment plant remained effective for one month of operations, made discontinuous by accidental power failures. After two days of very high daily share of air samples having patterns belonging to cluster 4 (namely May 18th (100% of e-nose patterns mapping on cluster 4) and May 24th 2017 (93%)), the discriminating ability of the e-nose was lost.

### 3.5. Projection of the External Dataset on the Map and Anomaly Confimation

**Figure 10:**
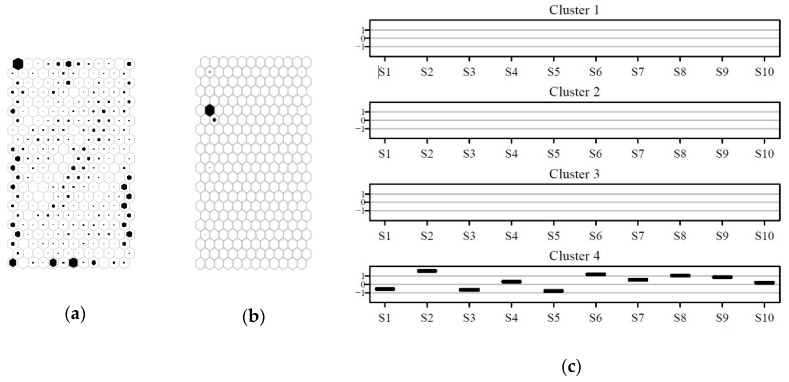
(**a**) Distribution of the building data on the map and (**b**) distribution of the external data on the map. The filling of each hexagon is proportional to the number of data mapping on the best matching unit (BMU) represented by the hexagon. (**c**) Interquartile ranges of the e-nose sensor responses for the external data set mapped on the SOM, grouped by cluster.

The external dataset (e-nose data collected from May 25th to June 28th) was suspected to have been generated either by a sensor failure or by a sudden change in the emission pattern of the industrial area. With the aim of checking variability of these data on the pattern variability represented by the SOM, they were projected onto the SOM model and all of them were found to have their BMU in cluster 4. The distribution of e-nose of the building dataset on the SOM prototypes is shown in Figure 10a, while the projection of external data on the map is shown in Figure 10b. The variability of sensor responses of external data projected on the clustered map is shown as interquartile boxes in Figure 10c. 

More than one month (May 25th to June 28th) of e-nose data from the external data set had extremely small variability, which is unlikely to occur in air monitoring, and were mapped on very few prototypes, all belonging to cluster 4, suggesting the occurrence of loss of dynamic response of the e-nose sensors for instrumental failure occurred on May 24th, and not detected by biweekly field calibrations.

## 4. Conclusions 

An environmental survey monitored ambient air for 83 days in the proximity of an industrial oil and gas pretreatment facility in Southern Italy, by means of a commercial e-nose equipped with ten MOS sensors, generating 80,017 vectors of ten sensor responses, acquired with a one-minute frequency. During the period from April 7^th^ to June 28^th^ 2017, power failures did not allow us to collect data for 11 whole days and occasionally for some hours. A photo ionization detector (PID) for measuring total volatile organic compounds TVOCs expressed as ppm_v_ of isobutylene, and a meteorological station completed the equipment of the monitoring site, providing ancillary data useful for studying ambient air contamination by gaseous compounds.

Compression and visualization of the information from the data set were obtained by building a self-organizing map model, whose prototype vectors were clustered by the k-means algorithm, with k selected by examining Davies–Bouldin indexes. An inspection of clustering results of SOM prototypes allowed us to evidence an anomaly in the functioning of the e-nose during the last 35 days of the survey, which had not been detected during field instrumental quality controls, pointing at the opportunity to a build a SOM model on the subset of the available data (37 days) with daily significant dynamic responses of the e-nose, before May 25th 2017. A map of 22 × 12 new prototype vectors was partitioned in four clusters, with different grades of e-nose responses. The e-nose vectors having best matching units in the fourth cluster of SOM prototypes show the highest sensor responses; TVOCs values collected simultaneously to these vectors had a relatively high median value (0.14 ppm_v_), while winds at these monitoring times had speeds with low median (<1 m/s, low dilution/dispersion of gases) and directions blowing more than 50% of the fourth cluster minutes from the area of the oil/gas pretreatment plant. Data from the last 35 days of the survey, excluded in the model building, were projected onto the SOM, but they were concentrated on a three prototype vectors of cluster 4, with very high prevalence of one of them. The very small variability of representations on the map of the 34,514 vectors collected from May 25th points at a substantial loss of dynamic responsivity of all of sensors of the e-nose, thus confirming/suggesting the occurrence of an instrumental collapse, possibly due to condensing of some vapor or ingression of semivolatile organic compounds in the measuring chamber.

Applications of e-noses in environmental monitoring at industrial sites proved to require thorough periodical field controls, for checking correct functioning of the multisensor systems out of the laboratory.

The proposed two step procedure allowed us to identify an anomaly in the multivariate space related to instrumental failure after 47 days from the beginning of the monitoring survey. The permanent change in the daily cluster share after May 24th, leading to a unique membership of all daily electronic nose data to cluster 3, points at the occurrence of a sudden critical event rather than to a progressive drift of responses of all the 10 sensors. The standardization of procedures for environmental applications of e-noses, implementing also the online warning system for system failures during a multi-month survey, is a needed step for supporting high frequency monitoring of air quality. The use of a self-organizing map algorithm followed by a cluster analysis of SOM prototypes to assess environmental data variability proved to be a helpful tool for sensor information assessment in this framework.

## Figures and Tables

**Table 1:** Sensor acronym used in the test and related features as reported by the manufacturer.

Acronym	Sensor Name	General Description	References	Signal^1^
S1	Aromatic 1	Aromatic compounds	Toluene, 10 ppm	G_0_/G
S2	Broad-range	Very sensitive, broad range sensitivity, react on nitrogen oxides, very sensitive	NO_2_, 1 ppm	G/G_0_
S3	Aromatic 2	Ammonia, used as sensor for aromatic compounds	Benzene, 10 ppm	G_0_/G
S4	Hydrogen	Mainly hydrogen, selectively, (breath gases)	H_2_ 100 ppb	G/G_0_
S5	Arom-aliph	Alkanes, aromatic compounds, less polar compounds	Propane, 1 ppm	G_0_/G
S6	Broad-methane	Sensitive to methane. Broad range, similar to No. 8	CH_3_, 100 ppm	G/G_0_
S7	Sulphur-organic	Reacts on sulfur compounds, H_2_S 0.1 ppm. Otherwise sensitive to many terpenes and sulfur organic compounds, which are important for smell, limonene, pyrazine	H_2_S, 1 ppm	G/G_0_
S8	Broad-alcohol	Detects alcohols, partially aromatic compounds, broad range	CO, 100 ppm	G/G_0_
S9	Sulph-chlor	Aromatics compounds, sulfur organic compounds	H_2_S, 1 ppm	G/G_0_
S10	Methane-aliph	Reacts on high concentrations >100 ppm, sometime very selective (methane)	CH3, 100 ppm	G/G_0_

^1^ G_0_ = baseline conductance; G = sample conductance.
